# Preclinical and clinical aspects of TNF-α and its receptors TNFR1 and TNFR2 in breast cancer

**DOI:** 10.1186/s12929-017-0398-9

**Published:** 2017-12-04

**Authors:** Isela Martínez-Reza, Lorenza Díaz, Rocío García-Becerra

**Affiliations:** 1 Departamento de Biología de la Reproducción, Dr. Carlos Gual Castro, Instituto Nacional de Ciencias Médicas y Nutrición Salvador Zubirán, Avenida Vasco de Quiroga No. 15, Col. Belisario Domínguez Sección XVI, C.P.14080 Ciudad de México, México; 2 Posgrado en Ciencias Biomédicas, Universidad Nacional Autónoma de México, Circuito Interior, Cuidad Universitaria, Av. Universidad 3000, 04510 Coyoacán, México D.F México

**Keywords:** TNF-α, TNFR1, TNFR2, Breast cancer

## Abstract

Breast cancer is the most common malignancy in women and a public health problem worldwide. Breast cancer is often accompanied by an inflammatory process characterized by the presence of proinflammatory cytokines such as tumor necrosis factor (TNF-α), which has important implications in the course of the disease. Inflammation has been described primarily as a favorable environment for tumor development. However, under certain conditions TNF-α can promote signals for activation, differentiation, survival or cell death, so the study of the variants of this cytokine, its receptors, the presence of polymorphisms and its implication in different phenotypes of breast cancer is necessary. Although the clinical application of TNF-α has been limited by its toxicity and side effects, preclinical and clinical studies have shown that these effects may partially be avoided via tumor-targeted delivery strategies. In this manner, TNF-α alone or combined with chemotherapy and radiotherapy can function as an adjuvant in the treatment of breast cancer.

## Background

Breast cancer ranks first in incidence and mortality in malignancies in women [1]. The classification of breast cancer based on the molecular phenotype depends on the presence of the estrogen receptor (ER), the progesterone receptor (PR), and/or the type 2 human epidermal growth factor receptor (HER-2). These molecular markers determine cancer behavior and response to therapy. Breast cancer is often accompanied by an inflammatory environment characterized by chronic inflammation involving the presence of immune cells (neutrophils, macrophages, B lymphocytes, plasma cells, CD4 Th1 lymphocytes), growth factors, mediator proteins (perforin, granzymes) and proinflammatory cytokines such as TNF-α [2, 3]. Controversial functions have been attributed to this latter molecule in breast cancer, including activation of apoptosis, tumor growth inhibition or promotion of tumor invasion, propagation and aggressive behaviour [4–7]. The TNF-α gene is located on chromosome 6p21.3 and encodes a membrane precursor molecule of 26 kDa (mTNF) [8]. This molecule is processed by a metalloprotease called TNF-α converting enzyme (TACE) that acts on Ala-66 and Val-67 amino acids of mTNF leading to the formation of a soluble protein (sTNF) of 17 kDa. Both TNF-α forms are biologically active [9]. TNF-α was originally identified as a mediator of tumor necrosis in the serum of animals treated with lipopolysaccharide, and is produced by immune cells, non-immune and tumor cells [10]. TNF-α is a cytokine that belongs to the TNF superfamily consisting of 19 proteins, it elicits various biological functions such as providing signals for activation, differentiation, survival and cell death. Also, it modulates the immune response and inflammation in multiple tissues and organs [11]. The effects of TNF-α are mediated through two different receptors, the gene encoding for receptor type 1 (TNFR1) is located on chromosome 12p13, giving rise to a 60 kDa protein. TNFR1 is also known as p55, p60, CD120a or TNFRSF1A [12]. The receptor type 2 (TNFR2) is encoded by the gene located on chromosome 1p36.2 and its mRNA translates into a protein of 80 kDa, also known as p75, p80, CD120b or TNFRSF1B [9, 13, 14]. Both receptors are transmembrane glycoproteins and belong to the TNF receptor superfamily with 29 members found in humans [11]. The two TNF receptors have similar extracellular domains consisting of multiple cysteine-rich repeats of approximately 40 amino acids in length, but different intracellular domains. TNFR1 is expressed in virtually all cell types except erythrocytes, while TNFR2 is found mainly in immune cells but it is also abundant in endothelial cells and hematopoietic lineage cells. TNF-α binds to both receptors with high affinity; showing a dissociation constant (Kd) of 2-5X10^-10^ and 3-7X10^-10^ for TNFR1 and TNFR2, respectively. Also, there are two soluble receptors (sTNFRs), generated by proteolysis of membrane bound receptors [15]. Soluble TNFRs are structurally identical to the extracellular binding domain of TNFR1 and TNFR2 [16]. The TNFR1 is activated through both soluble and membrane TNF-α, whereas TNFR2 is mainly activated by mTNF-α [17]. Most biological effects of TNF-α such as cytotoxicity and proliferation occur via TNFR1 activation [18]. In contrast to TNFR2, the intracellular region of TNFR1 contains a death domain (DD) which has been associated with TNF-α-mediated cytotoxicity [19]. Depending on the cellular context, conditions and microenvironment, TNFRs activation may lead to the induction of proliferation, apoptosis or necroptosis. Activation of such different cellular responses reflects the existence of a complex regulatory network after receptor activation. Molecular mechanisms of TNF-α signaling are reviewed in detail elsewhere [20].

### Preclinical aspects of TNF-α and their receptors in breast cancer

A great deal of preclinical research has been undertaken in order to understand the relationship between TNF-α and breast cancer development, progression or as a therapeutic option. In this regard, some contradictory evidence has shown both growth inducing or antitumorigenic effects of this cytokine in the context of breast cancer. First, we will compile the evidences supporting a protumorigenic effect of TNF-α.

TNF-α promoted growth, migration and invasion of MCF-7 estrogen receptor (ER)- positive and MDA-MB-231 (triple negative) breast cancer cell lines, partially by inducing the expression of matrix metalloproteinases (MMPs) and dipeptidylpeptidases [4, 5]. Likewise, TNF-α promoted the growth of MDA-MB-231 and SKBR-3 (HER-2 positive) cell lines by increasing the oncoprotein hepatitis B X interacting protein (HBXIP). Mechanistically, TNF-α activates TNFR1 forming a positive feedback loop of TNFR1/NF-κB (and/or p38)/p-STAT3/HBXIP/TNFR1 [21]. In a lung metastasis model of murine breast cancer, TNF-α-activated mesenchymal stromal cells (MSCs) significantly enhanced tumor metastasis via CXCR2+ neutrophil recruitment [22]. Moreover, the anti-TNF-α therapy using infliximab, a monoclonal antibody that precludes both sTNF-α and mTNF-α from binding to their receptors, suppressed the migration and invasion of MDA-MB-231 cells while inhibited bone metastases in an in vivo model [23]. This may be linked to the evidence showing that TNF-α has an important implication in the course of breast cancer development, which could be the result of its ability to regulate signaling pathways involved in gene expression regulation. In this regard, microRNAs (miRNAs), an important class of small endogenous noncoding RNAs (21–25 nucleotides long) that post-transcriptionally regulate gene expression, are known to have an important role in carcinogenesis, and particularly, miRNA-23b and miRNA-27b have been associated with poor prognosis in human breast cancer. In fact, the suppresion of miR-23b/27b activity upregulates Nischarin, a tumor suppressor of breast cancer [24]. Interestingly, TNF-α downregulated Nischarin and stimulated the expression of both miRNAs in HER2 and triple negative breast cancer cell lines. The expression of both miRNAs is stimulated by TNF-α through the AKT/NF-κB signaling pathway [25]. On the contrary, the overexpression of the miRNAs miR-509 and miR29 has shown to increase apoptosis, inhibit cell proliferation and invasion via suppression of TNF-α or inhibition of TNFR1 expression in triple-negative and ER positive breast cancer cells, suggesting a pro-tumorigenic effect of TNF-α or its receptor in this kind of tumors [26, 27]. On the other hand, mTNF expressed by lymphoma cells protected them from apoptosis by inducing constitutive activation of NF-κB. In order to study the differential bioactivities of sTNF and mTNF, a monoclonal antibody (mAbTNF-α) that binds both the full-length and the N-terminal truncated fragments of mTNF was developed. Importantly, this antibody does not cross-react with sTNF. In a mouse model of breast cancer, mAbTNF-α administration caused retardation of tumor growth and in some cases complete tumor regression, suggesting that mTNF is involved in tumor survival [28]. Moreover, the expression of the adhesion molecule CD44v6, a variant of CD44 known to promote cell migration and survival during cancer metastasis, was inhibited by mAbTNF-α administration [29].

Furthermore, it has been demonstrated in MDA-MB-231 cells, that TNF-α increased MMP-9 expression and activity by inducing AP-1 DNA binding activity, thus reinforcing the concept of a protumorigenic effect of TNF-α in breast cancer. Indeed, the MMPs are a major group of enzymes that regulate cell-matrix composition, and in particular MMP-9 is involved in angiogenesis as well as tumor growth, invasion and metastasis of various tumors, including breast cancer. [30]. Another cancer-related process that involves TNF-α activity is the epithelial-mesenchymal transition (EMT). This is a process in which epithelial cells lose their phenotypic characteristics and acquire mesenchymal properties such as loss of E-cadherin (epithelial marker) and induction of vimentin (mesenchymal marker), loss of cell adhesion, disruption of cell-cell junctions and extensive reorganization of the actin cytoskeleton, resistance to apoptosis, increased mobility and invasiveness. EMT is carried out during embryogenesis, carcinogenesis, metastasis and tumor recurrence [31]. Interestingly, it has been described that conditioned media from TNF-α-primed stem cells from human adipose tissue (hASCs) may induce EMT on MCF-7 cells by reducing E-cadherin and increasing vimentin expression. This EMT process was accompanied by increased invasion, migration, and urokinase type-plasminogen activator (uPA) expression and was mediated by increased expression of transforming growth factor-β1 (TGF-β1). These data suggested that TNF-α primed hASCs might enhance the malignancy of MCF-7 cells, highlighting potentially important implications of TNF-α in breast cancer progression and dissemination [32].

MUC16, also knows as as the antigen CA125, is a transmembrane mucin that protects tumor cells from the immune system promoting resistance to various chemotherapeutic agents. MUC16 is used as tumor marker for some human cancers including breast cancer [33]. It has been shown that TNF-α and/or IFN-γ stimulated MUC16 mRNA levels in a dose dependent manner in ER positive breast cancer cells. In fact, combined treatment with both citokines resulted in a significant increment of MUC16 expression when compared to either molecule alone [34]. The increase of MUC16 expression by TNFα and IFNγ is thought to be mediated through NFκB and PPARγ modulation [34, 35].

**Fig. 1:**
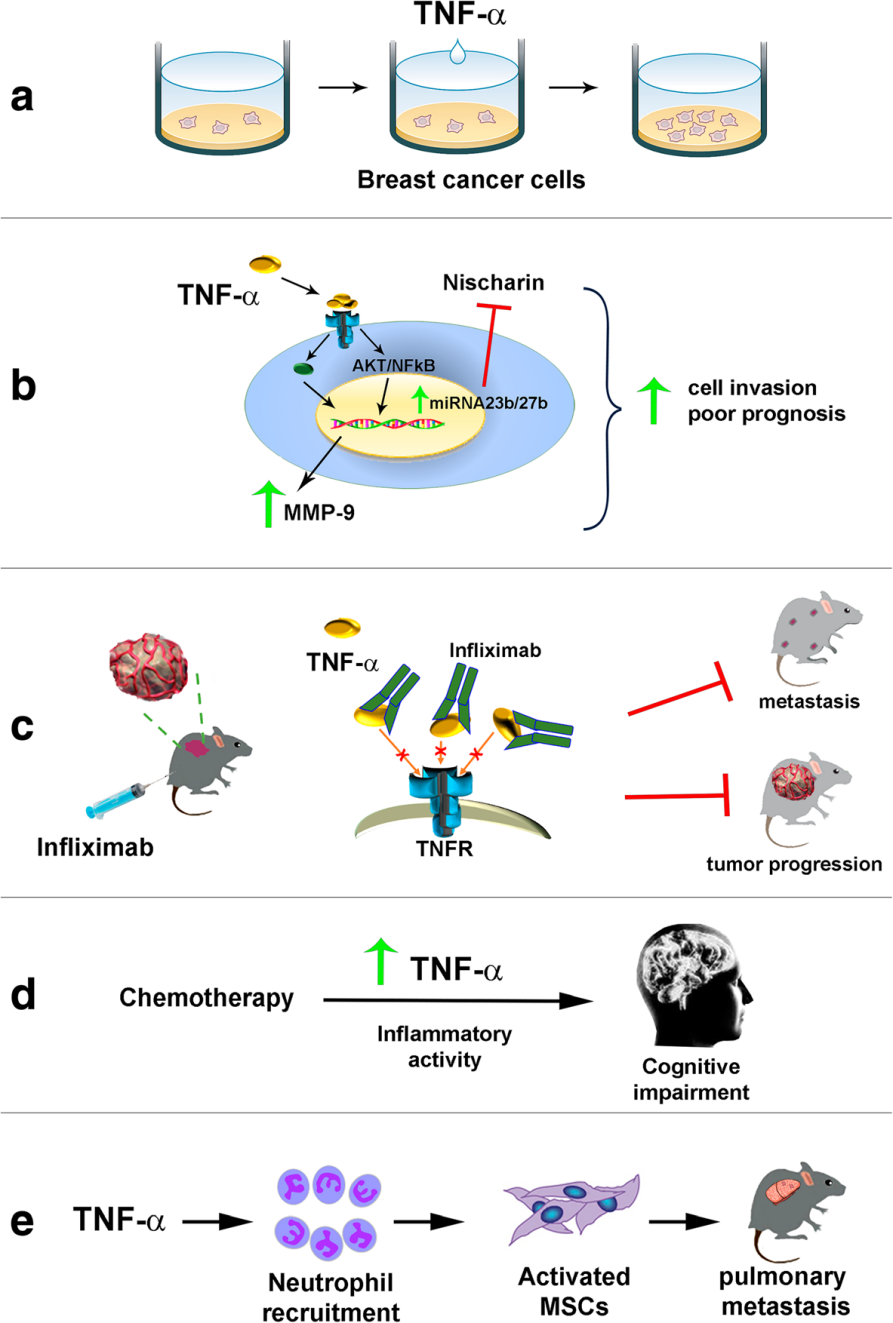
Protumorigenic effects of TNF-α in breast cancer. **a** TNF-α promotes growth, migration and invasion of diverse breast cancer cell lines (4,5,21). **b** TNF-α stimulates the expression of miRNA-23b and miRNA-27b through the AKT/NF-κB signaling pathway, inhibiting Nischarin, a suppressor of tumor growth. TNF-α also stimulates the expression of MMP-9. All of this is associated with poor prognosis and cell invasion in HER2 and triple negative breast cancer cells (24, 29). **c** Blocking m/sTNF-α activity with the antibody infliximab directed against TNF-α receptor (TNFR) slows tumor growth, induces tumor regression and inhibits bone metastases in mice, highlighting TNF-α tumorigenic effects (23). **d** Chemotherapy-induced cognitive impairment might result from TNF-α inflammatory activity (57). **e** TNFα-activated mesenchimal stromal cells (MSCs) enhanced tumor metastasis via neutrophil recruitment (22)

Finally, TNF-α gene knockout has shown to induce apoptosis and inhibit cell proliferation in the triple negative breast cancer cell line Hs578T [36]. A summary of TNF-α protumorigenic effects is found in Fig. 1.

On the other hand, there are also compelling evidences of antineoplastic effects of endogenous and exogenous TNF-α, which are described below.

Although mTNF is the precursor molecule of sTNF, the bioactivities of both forms are different. Indeed, a previous study showed that mTNF was able to kill more tumor-derived cells than sTNF, including those from leukemia, hepatocarcinoma, lymphoma, fibrosarcoma and breast cancer. The cytotoxic effects induced by mTNF and sTNF were mainly apoptosis and necrosis, respectively [28]. Moreover, TNF-α showed cytotoxic effects and induced apoptosis in MCF-7 and Hs578T breast cancer lines. Interestingly, the vitamin D analogue CB1093 augmented TNF-α-induced cytotoxicity [37]. In this regard, high circulating levels of insulin like-growth factor I (IGF-I) increased the risk of breast cancer, while TNF-α prevented IGF-I-dependent DNA synthesis, leading cells into G0/G1 phase and avoiding them from entering the S phase of the cell cycle in MCF-7 cells [7]. Of note, Burow et al. showed differential susceptibility to apoptosis induced by TNF-α in MCF-7 cells obtained from different laboratories, probably due to differences in TNFR expression or other factors which may explain the divergent results of this cytokine in this cell line [38].

TNF-α as an antitumor drug is currently being tested, and even if its clinical use has been limited due to systemic toxicity, low doses of a TNF-α derivative that targets tumor neovessels have shown reduced toxicity while synergized antitumor-activity of doxorubicin and melphalan, cisplatin, paclitaxel, and gemcitabine in murine lymphoma, fibrosarcoma, and mammary adenocarcinoma models [39]. Likewise, TNF-α increased the cytotoxicity of chemotherapy (docetaxel, 5-fluorouracil, cisplatin) and radiotherapy against breast cancer in in vitro and in vivo studies [40].

**Fig. 2:**
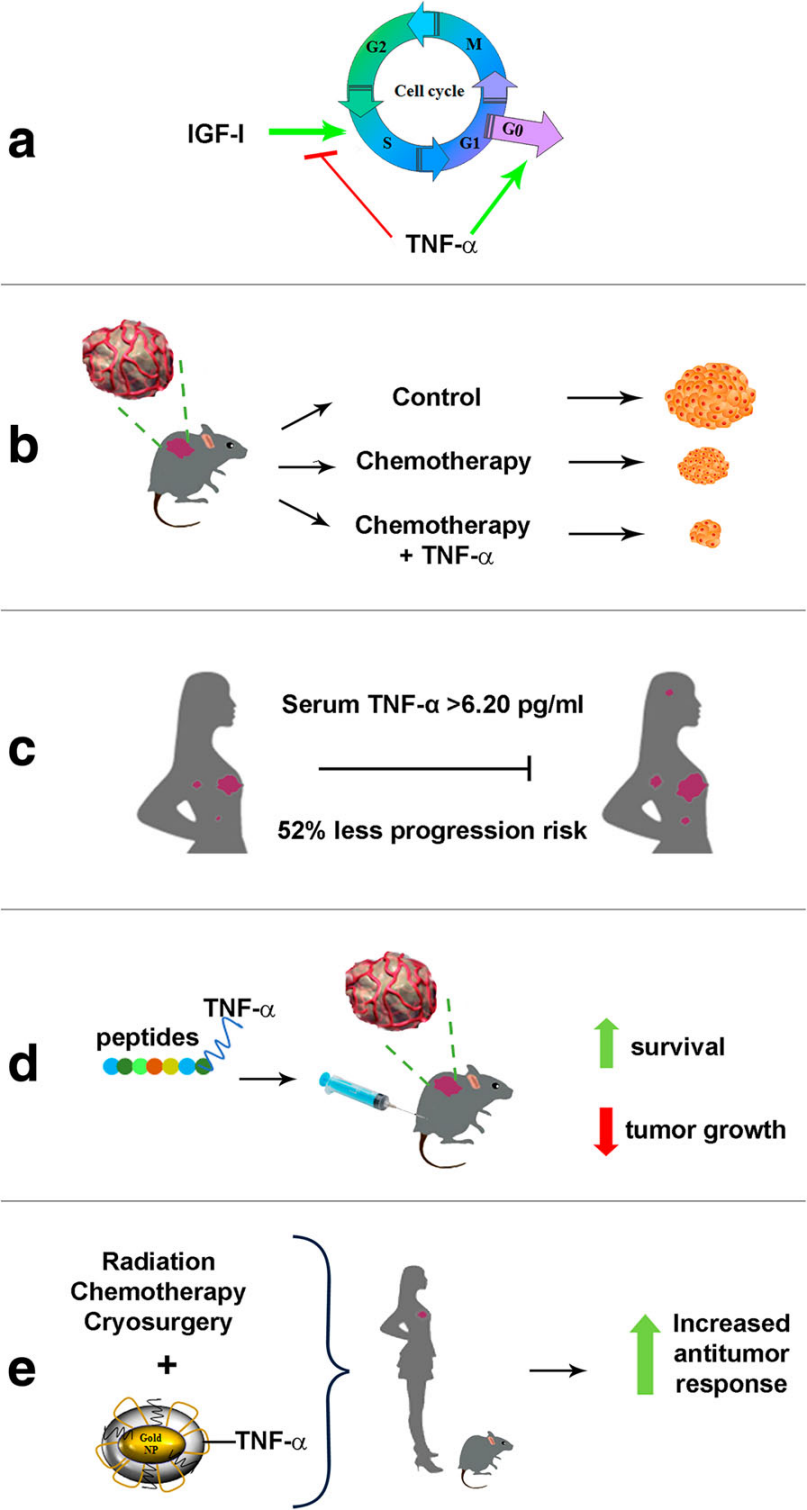
Antitumorigenic effects of TNF-α in breast cancer. **a** TNF-α impairs cell cycle progression, preventing IGF-I-dependent DNA synthesis and arresting cells in G0/G1 phase in ER positive breast cancer cells (7). **b** Administration of tumor vasculature-targeted TNF-α synergizes chemotherapy in models of mammary adenocarcinoma (38). **c** TNF-α > 6.20 pg/ml is associated with 52% less risk of progression of breast cancer (54). **d** The use of tumor-homing peptides fused to TNF-α results in increased anti-tumor activity (47). **e** Nanoparticles-coupled TNF-α improve response to radiation, chemotherapy and cryosurgery, resulting in increased antitumor response (40–44)

Several targeted delivery methods with minimal invasion have been developed for TNF-α systemic administration, as is the case of CYT-6091 nanoparticles which were constructed by simultaneously binding TNF-α and thiolyated polyethylene glycol to the surface of colloidal gold particles [41, 42]. Nanoparticles (<100 nm) have successfully been used to deliver therapeutic drugs specifically targeting cancer cells and confining the treatment mainly within tumors, with the advantage of shortening the patient’s recovery time and causing less morbidity and fewer complications [43]. Data indicated CYT-6091 (30 nm) preferential uptake in cancer models of colon, breast and prostate. Similarly, increased CYT-6091 accumulation in tumor tissue was found compared to normal tissues over time [44]. In fact, gold nanoparticles >10 nm were not detected in blood, liver, spleen, kidney, testis, thymus, heart, lung or brain [45]. Besides, CYT-6091 used in combination with hyperthermia or cryosurgery resulted in a synergistic antitumor response when compared with the monotherapy. Indeed, the combination of CYT-6091 with cryosurgery achieved complete tumor destruction [46]. Furthermore, in a murine breast tumor model, CYT-6091 combined with high-dose radiation therapy was effective in reducing both tumor interstitial fluid pressure and growth, and was associated with marked vascular damage which overall resulted in a synergistic antitumor response [47]. This was mainly due to the vascular-targeting properties of the compound, as seen when neoangiogenic vessels are specifically reached using a Cys-Asn-Gly-Arg-Cys peptide-TNF-α fusion product that enhances lymphocyte infiltration in tumors, resulting in increased therapeutic potential of the immunotherapy. This tumor-homing peptide recognizes an aminopeptidase N (CD13) isoform selectively expressed by endothelial cells in tumor vessels [48]. Overall, preclinical data using tumor-vasculature-targeted TNF-α has shown improved antitumoral effects of chemotherapeutic agents, obtaining promising results for breast cancer therapy. In this regard, CYT-6091 has already passed through phase 1 trials. A summary of TNF-α antitumorigenic effects is found in Fig. 2.

### Clinical aspects of TNF-α and their receptors in breast cancer

While in the serum of healthy women TNF-α is generally not detected, clinical studies have reported high levels of this cytokine in patients with breast cancer [49, 50]. Likewise, high levels of mTNF-α expression has been found in primary breast cancer tumors, lower levels in hyperplasias, and undetectable levels in normal breast tissue [28]. In this regard, breast cancer is a complex disease with different stages and molecular signatures, making it difficult to understand the role of TNF-α in this pathology [51]. To date, it is unknown whether the origin of TNF-α surrounding the tumor is produced and released by cancer cells as an evasion strategy to the host immune response or whether it is secreted by cells of the immune system in order to boost the host immune response to stop tumor cells from growing and eventually eliminate the tumor [52–54]. However, some evidences suggest that the final response depends on many factors, such as the levels of expression of TNF-α and/or its receptors. For example, in a cohort of metastatic breast cancer patients treated with chemotherapy, it was shown that a serum TNF-α concentration > 6.20 pg/ml was associated with 52% less risk of breast cancer progression [55]. Also, in naïve breast cancer patients, high serum sTNFRs levels were found together with a trend toward higher plasma concentrations of TNF-α compared to controls. Interestingly, after 3 months of treatment with hormonal therapy or chemotherapy, sTNFRs expression remained constant, but TNF-α levels decreased [56, 57]. In addition, TNFR1 was found significantly increased in human breast cancer tissues and the breast cancer cell lines MCF-7, MDA-MB-231 and T47D when compared against non-tumor tissues [27].

Noteworthy, chemotherapy-induced cognitive impairment, a common sequelae of cancer therapy, has been suggested to result from increased inflammatory mediators such as TNF-α. Indeed, survivors of breast cancer frequently show significant cognitive damage, and notably, findings from a study with breast cancer patients showed evidence of TNF-α and IL-6-mediated altered hippocampal volume and verbal memory difficulties following chemotherapy [58]. Therefore, some efforts have been performed to neutralize TNF-α signaling during chemotherapy. By instance, a study with breast cancer and non-Hodgkin lymphoma patients evaluated the coadministration of doxorubicin with the anti-oxidant mesna, finding that the combined regimen was able to reduce the levels of chemotherapy-induced TNF-α, TNFR and related cytokines [59]. Further studies should be conducted in order to evaluate serum TNF-α and their receptors abundance in distinct breast cancer subtypes before and after conventional treatment.

Interestingly, a factor that contributes significantly to breast cancer susceptibility is the presence of a polymorphism of TNF-α (TNF-α-308) located in the promoter region of the gene, that involves the substitution of a guanine by an adenine, as reported in caucasian and mexican women [60, 61]. This change affects TNF-α gene expression, increasing its production. Moreover, the presence of TNF-α-308 was related to vascular invasion in breast cancer [62]. In addition, bi-allelic polymorphisms of TNFR2 have been reported (located in exons 4, 6, 9 and 10). In particular, the variation in exon 6 results in the substitution of methionine by arginine in codon 196 (196 M); the altered amino acid is found in the extracellular region of the receptor responsible for proteolytic cleavage, producing the soluble form of TNFR2 [63]. This 196 M/R-TNFR2 variation has been significantly associated with breast carcinoma, with particular importance in post-menopausal patients, considering this a factor for the late onset of breast cancer. On the other hand, the presence of 196R–TNFR2 in patients with breast carcinoma has been associated with increased overall survival and disease-free survival compared with the absence of the 196R allele; whereby the detection of this polymorphism can serve as a marker for predicting relapse and death of patients [64]. In diseases characterized by chronic inflammation such as: ankylosing spondylitis, Crohn’s disease, juvenile idiopathic arthritis, psoriatic arthritis, rheumatoid arthritis and ulcerative colitis, the use of TNF-α inhibitors (TNFis), such as adalimumab, certolizumab, etanercept, golimumab and infliximab, has shown a good therapeutic outcome. Nevertheless, in the context of breast cancer, clinical studies have suggested that the use of TNFis to block TNF-α signaling, rather than reducing inflammation may increase the risk of malignancy by promoting invasiveness and tumor growth, which deserves further investigation. To date, there are no conclusive results indicating therapeutic benefit in cancer patients treated with TNFis; however, it has been shown that its use did not increase the risk of malignancy in breast tumors [53].

In general, the clinical application of TNF-α in breast cancer patients has been restricted because of mild systemic toxicity. Some reported symptoms in such patients include fever, chills, hypotension, fatigue, anorexia, headaches, reduced hippocampal volume, verbal memory difficulties and impaired metabolism of triglycerides [58, 65]. In order to overcome these unwanted secondary effects of TNF-α, the targeted delivery strategy using CYT-6091 has been tested in a phase I clinical trial in advanced stage cancer patients including breast ductal carcinoma and plans for a phase II trial are ongoing [41, 47]. In the phase I clinical trial it was demonstrated that the doses of 50 to 600 micro g/m^2^ of recombinant human TNF-α, previously shown to be toxic, presented a safe toxicity profile when using the CYT-6091 nanoparticle delivery approach. Moreover, nanoparticles trafficked to the tumor, but not to healthy tissue, highlighting its tumor-targeting ability [41, 45].

## Conclusions

TNF-α is a proinflammatory cytokine with opposing effects on breast cancer cells. TNF-α, through a complex regulatory network after activating its receptors, can induce apoptosis or necroptosis, cell growth, invasion or propagation of cancer cells. Controversies in response to TNF-α may be due to the complexity of the signaling pathways triggered by this cytokine, the different levels of m/sTNF-α, m/sTNFR 1 and 2, together with the presence of polymorphisms on the cytokine and its receptors. Also, the cellular context, conditions and microenvironment play a role in TNF-α signaling and final biological effects. Interestingly, in preclinical and clinical studies in breast cancer, TNF-α has shown important antitumor activity alone and in combination with chemotherapy, radiation therapy and cryosurgery. Although the clinical application of TNF-α has been restricted because of its toxicity, the use of tumor-targeted therapeutic strategies employing nanoparticles or tumor-homing peptides offer great promise for the treatment of breast cancer.

## Abbreviations

196 M: Arginine in codon 196
AKT: Protein kinase B
Arg: Arginine
Asn: Asparagine
CD13: Aminopeptidase N
Cys: Cisteine
CYT-6091: nanoparticle binding TNF-α and thiolyated polyethylene glycol to the surface of colloidal gold particle
DD: Death domain
EMT: Epithelial-mesenchymal transition
ER: Estrogen receptor
Gly: Glycine
hASCs: TNF-α-primed stem cells from human adipose tissue
HBXIP: Oncoprotein hepatitis B X interacting protein
HER-2: The type 2 human epidermal growth factor receptor
IGF-I: Insulin like-growth factor I
KDa: Kilodaltons
mAbTNF-α: Monoclonal antibody TNF
miRNAs: MicroRNAs
MMP: Metalloproteinase
mTNF: Membrane TNF
MUC16: Mucin 16
NF-κB: Nuclear factor κB
PR: Progesterone receptor
STAT: Signal transducers and activators of transcription
sTNF: Soluble TNF
sTNFRs: Soluble TNF-α receptors
TACE: TNF-α converting enzyme
TGF-β: Transforming growth factor-β
TNFis: TNF-α inhibitors
TNFR1: TNF-α receptor type 1
TNFR2: TNF-α receptor type 2
TNF-α: Tumor necrosis factor
TNF-α-308: Polymorphism 308 of TNF-α
uPA: Type-plasminogen activator
